# Properdin Levels in Human Sepsis

**DOI:** 10.3389/fimmu.2015.00024

**Published:** 2015-02-02

**Authors:** Cordula M. Stover, John McDonald, Simon Byrne, David G. Lambert, Jonathan P. Thompson

**Affiliations:** ^1^Department of Infection, Immunity and Inflammation, College of Medicine, Biological Sciences and Psychology, University of Leicester, Leicester, UK; ^2^Department of Cardiovascular Sciences, Division of Anaesthesia, Critical Care and Pain Management, University of Leicester, Leicester, UK

**Keywords:** sepsis, complement, properdin, intensive care, recovery

## Abstract

Properdin is a normal serum protein that increases the production of complement activation products by binding C3b integral to convertase complexes and amplifying their activity at the site of activation. Thereby, it not only can aid in the resolution of infection but also contribute to tissue damage. In human sepsis, circulating complement C3 concentrations are decreased, though C3 is described as a positive acute phase reactant. However, properdin levels in human sepsis have not been reported. In this study, serum from 81 critically ill patients (predominately abdominal and respiratory sepsis) were analyzed for properdin levels at defined points of their stay in the intensive care unit (ICU) and compared with 61 age and sex-matched healthy volunteers. Properdin concentrations were significantly decreased in patients with sepsis on admission to ICU, but increased after clinical recovery to exceed levels observed in healthy volunteers. Properdin concentrations at ICU admission were decreased in non-survivors of sepsis compared to survivors, but this did not correlate with APACHE II score. However, pathologically low properdin levels (<7 μg/ml) were related to increased duration of treatment.

## Introduction

Sepsis is a life threatening condition in which a systemic inflammatory reaction may progress to shock, hypoperfusion of lungs, kidneys, and intestines and via consumption of coagulation factors to intravascular coagulopathy. Cytokine storm, systemic macrophage activation, complement activation, and activation of the coagulation cascade are factors that drive the organism from a state of overinflammation to immune exhaustion. The complement system is primarily a humoral, hierarchically arranged, system in which activation of recognition molecules is translated to enzymatic activity of protein complexes, which cleave C3 and C5 to generate potent anaphylatoxins, C3a and C5a. The strength of activation also determines cell activities, such as phagocytosis, respiratory burst, and vascular leakage. The contribution of complement activation to tissue damage in septic conditions is thought to be so significant that attempts are made to develop therapeutic blockers in order to rein in its activity (1). However, because complement operates over a wide range of biological functions in health and disease, choosing the window of opportunity in a polymorphic system (2) is likely to be very difficult. In experimental animal models of sepsis, however, treatment with anti-C5a or anti-C5aR antibodies in clear relation to the defined onset of sepsis has proven efficacious (3, 4).

The role of low levels of mannan binding lectin (MBL), one of the lectin pathway recognition molecules, as a single determinant in the outcome from severe shock is not clear; while studies link low MBL levels to an increased risk of developing the systemic inflammatory response syndrome, others assign a beneficial effect to low MBL levels because of the potentially reduced pro-inflammatory reaction (5). A prospective study of septic patients in comparison with healthy controls found depressed levels of complement C3 and elevated complement activation products C3a, Factor Bb and C4d, during the first 2 days of intensive care treatment (6). The observed increase in C5a in septic samples is consistent with previous work analyzed by the Bayesian inference approach (6). However, in sepsis, significant amounts of C5aR are found in serum, most likely shed from neutrophils (7); properdin is produced by neutrophils (8). Properdin is the only amplifier of complement activation but there are no data on properdin levels in human sepsis. A genetic deficiency of properdin predisposes to death from meningococcal septicemia in man (9), and in mice, significant impairment of survival is observed in endotoxin shock (10).

The aim of this study was to investigate properdin concentrations in critically ill patients admitted to the intensive care unit (ICU) with sepsis, using serum samples taken during a recently published observational study (11).

## Materials and Methods

Patients admitted to ICU at Leicester Royal Infirmary with a diagnosis of sepsis, and healthy volunteers matched for age and sex were recruited, as previously described (11). Permission to measure properdin in these samples was granted by University Hospitals of Leicester NHS Trust (R&D reference: UHL 11348 “Properdin and inflammatory biomarkers in sepsis”). Patients’ samples were taken on Days 1 and 2 of ICU admission, and a further sample was obtained after clinical recovery from sepsis. Archived, anonymized serum samples from 81 patients (40 m/41 f; 19-87 years) and of 61 age and sex-matched volunteers (29 m/32 f; 21-85 years) were analyzed. The origin of sepsis was as follows: abdominal *n* = 41, respiratory *n* = 25, neutropenic *n* = 5, urosepsis *n* = 2, and a small group of *n* = 8 made up of line infection (*n* = 2), fasciitis (*n* = 4), postpartum sepsis (*n* = 1), and abscess (*n* = 1).

Properdin was determined from 1/5000 serum sample dilutions using commercial kits (Hycult Biotech, Uden, The Netherlands), following the manufacturer’s protocol. Concentrations from duplicate measurements were calculated from a standard curve set up in duplicate. Those below the lowest dilution of the standard curve (cut off 0.3 ng/ml) were classed as deficient (zero).

### Statistical analysis

Data for properdin concentrations were normally distributed and were analyzed by an unpaired *t*-test between two groups. Duration of ICU or hospital stay was not normally distributed and was compared against properdin concentrations by non-parametric two-tailed Spearman correlation. *P*-values <0.05 were considered statistically significant.

## Results and Discussion

Samples were available for 81, 66, and 45 patients on Days 1, 2 and recovery, respectively. Clinical and other data have been reported previously (11). While consecutive levels were significantly lower at Days 1 and 2 of ICU admission compared to matched volunteers, levels increased significantly at clinical recovery (Table 1). Properdin levels did not correlate with survival time in those who succumbed within 1-3 days after admission to ICU. There was no relationship between properdin and total white cell or neutrophil counts. Properdin levels were significantly higher after clinical recovery compared to healthy volunteers. At the timepoint of clinical recovery, however, there was still biochemical evidence of inflammation (CRP and IL-8 concentrations had not completely normalized; increased leukocyte, neutrophil and platelet counts compared to the control group) (11). Properdin levels in the healthy volunteers of European background analyzed as part of this study were higher than those reported in a recent study for healthy South East Asians (12).

**Table 1:** **Properdin levels in sera of critically ill patients with sepsis admitted to ICU and a healthy control group**.

Group	Range (μg/ml)	Mean ± SD
Day 1 (*n* = 81)	0–38.8	9.0±7.6
Day 2 (*n* = 66)	0–30.9	8.9±6.9
Clinically recovered (*n* = 45)	2.4–51.2	22.9±11.1 ^a^^,^^b^
Volunteers (*n* = 61)	7.6–34.10	18.4±5.5^c^
Day 1, survivors (*n* = 60)	0–38.8	9.8±8.1
Day 1, non-survivors (*n* = 21)	0–21.2	6.8±5.2

*The time point of clinical recovery from sepsis was variable*.

*^a^*p* < 0.0001 against Days 1 and 2*.

*^b^*p* < 0.0008 against volunteers*.

*^c^*p* < 0.0001 against Days 1 or 2; Day 1 vs Day 2 n.s*.

The overall 30-day mortality in patients was 21/81 (25.9%). For four patients, in whom there (reproducibly) was no antigenically detectable properdin in their initial serum samples, no subsequent sample existed to exclude a genetic deficiency. But, as properdin is an x-chromosome linked gene and a primary deficiency uncommon, the possibility of a gene defect does not appear likely in the case of the two female patients. When comparing properdin levels determined for samples obtained on day 1 of admission to ICU between survivors and non-survivors, properdin appeared to be lower in non-survivors but this did not reach statistical significance (Table 1).

**Figure 1:**
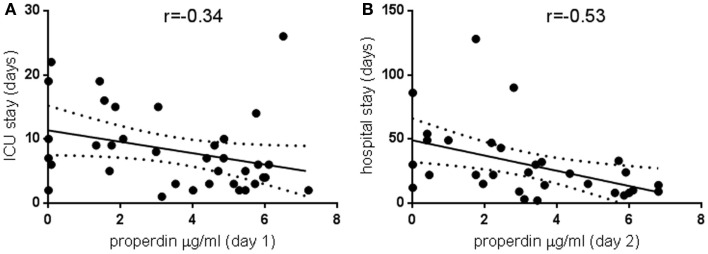
**Pathologically low properdin levels in the critically ill and the relationship to treatment duration**. Properdin levels lower than 7 μg/ml, the lowest of the normal range established in this study, were plotted against duration of intensive care treatment **(A)** 36 pairs, *p* < 0.05, and total duration of hospitalization **(B)** 31 pairs, *p* < 0.005. Spearman correlation coefficients are indicated; regression lines and 95% confidence bands of the best-fit line are shown.

Properdin levels determined on day 1 of ICU admission did not relate to APACHE or SOFA scores (data not shown) or duration of stay in ICU or in hospital, but for the whole group, the ICU stay correlated significantly with hospital stay (80 pairs analyzed, *r* = 0.6, *p* < 0.0001). In other studies, depressed C3 levels found in sepsis were not related to mortality or complications such as pneumonia and hemorrhage (13), but decreased C3 levels were linked to increased hospital stay (14). Therefore, a subgroup analysis was performed for very low properdin levels. These were defined as <7 μg/ml, the lower end of the normal range established in this study. Pathologically low properdin levels on Days 1 and 2 negatively correlated with the duration of intensive care treatment and overall hospitalization, respectively (Figures 1A,B).

There are two possible interpretations for these findings: (i) the decline in properdin levels is a descriptor of severity of sepsis due to its ability to bind to LPS of different pathogens (15) or to damaged cells (16) and (ii) properdin is involved in the pathophysiological mechanisms of sepsis.

Recent data indicate that the likely determinants of outcome from sepsis are the cell phenotypes, which emanate from this condition in dependence of the level of complement components. Depressed serum C3 levels have been linked to the emergence in the critically ill of an immune suppressive T_reg_ population (14). So, altered complement levels may be a mirror of changing cell activities selected during the septic immune response (17). Alternatively, a decline in circulating properdin levels may signify increased complement usage in septic end organs (18) or on inflamed endothelium (19).

## Conflict of Interest Statement

The authors declare that the research was conducted in the absence of any commercial or financial relationships that could be construed as a potential conflict of interest.
